# Cysteine Oxidations in Mitochondrial Membrane Proteins: The Case of VDAC Isoforms in Mammals

**DOI:** 10.3389/fcell.2020.00397

**Published:** 2020-06-04

**Authors:** Simona Reina, Maria Gaetana Giovanna Pittalà, Francesca Guarino, Angela Messina, Vito De Pinto, Salvatore Foti, Rosaria Saletti

**Affiliations:** ^1^Section of Molecular Biology, Department of Biological, Geological and Environmental Sciences, University of Catania, Catania, Italy; ^2^Section of Biology and Genetics, Department of Biomedical and Biotechnological Sciences, University of Catania, Catania, Italy; ^3^Organic Mass Spectrometry Laboratory, Department of Chemical Sciences, University of Catania, Catania, Italy

**Keywords:** cysteine overoxidation, outer mitochondrial membrane, voltage-dependent anion selective channel isoforms, Orbitrap Fusion Tribrid, posttranslational modification, ROS

## Abstract

Cysteine residues are reactive amino acids that can undergo several modifications driven by redox reagents. Mitochondria are the source of an abundant production of radical species, and it is surprising that such a large availability of highly reactive chemicals is compatible with viable and active organelles, needed for the cell functions. In this work, we review the results highlighting the modifications of cysteines in the most abundant proteins of the outer mitochondrial membrane (OMM), that is, the voltage-dependent anion selective channel (VDAC) isoforms. This interesting protein family carries several cysteines exposed to the oxidative intermembrane space (IMS). Through mass spectrometry (MS) analysis, cysteine posttranslational modifications (PTMs) were precisely determined, and it was discovered that such cysteines can be subject to several oxidization degrees, ranging from the disulfide bridge to the most oxidized, the sulfonic acid, one. The large spectra of VDAC cysteine oxidations, which is unique for OMM proteins, indicate that they have both a regulative function and a buffering capacity able to counteract excess of mitochondrial reactive oxygen species (ROS) load. The consequence of these peculiar cysteine PTMs is discussed.

## Genome–Proteome Discordance: an Overview on Protein Posttranslational Modifications

The mammalian proteome is vastly more complex than the related genome: an imbalance between one million different proteins against approximately 25,000 genes is estimated in humans (International Human Genome Sequencing Consortium, 2004; Clamp et al., 2007). The reasons for this inconsistency reside both in the molecular mechanisms that allow a single gene to encode for numerous proteins (i.e., alternative splicing, genomic recombination, alternative promoters, and termination sites) and in the posttranslational modifications (PTMs) which represent a wide range of chemical changes that proteins may undergo after synthesis. They include the specific cleavage of protein precursors, the covalent addition or removal of low-molecular groups, and the formation of disulfide bonds or other redox modifications (Wang et al., 2014; Duan and Walther, 2015). PTMs are crucial for several cellular processes such as protein turnover and signaling and are commonly mediated by enzymatic activity. Many of them have been extensively characterized, and phosphorylation is perhaps the most known example. Conversely, only in the last two decades have reactive oxygen species (ROS)/reactive nitrogen species (RNS) been identified as physiological regulators of intracellular signaling pathways through the covalent modification of specific cysteine residues within redox-sensitive proteins (Chung et al., 2013). Although the “oxidative posttranslational modifications” (Ox-PTMs) still represent a little explored field, the increasing number of tools aimed at identifying and quantifying them [e.g., high-throughput mass spectrometry (MS) analysis] continues to broaden the knowledge of redox regulation (Spickett and Pitt, 2012; Shakir et al., 2017; Anjo et al., 2019).

### Peculiarity of Cysteine Ox-PTMs and Their Correlation With Pathological Conditions

Because of their redox-reactive thiol (–SH) side chain, cysteine residues are likely subjected to various Ox-PTMs including *S*-nitrosylation (or *S*-nitrosation, SNO), sulfhydration (SSH), *S*-acylation, *S*-glutathionylation, disulfide bonds (RS-SR), sulfenylation (SOH), sulfinic acid (SO_2_H), and sulfonic acid (SO_3_H) (Forrester and Stamler, 2007; Murray and Van Eyk, 2012; Alcock et al., 2018). Except for sulfonic acid, all the reported Ox-PTMs are readily reversible and ruled by specific enzymatic activities. Sulfiredoxin (Srx1), for example, acts on oxidative states up to sulfinic acid by reducing them back to thiol in an ATP-dependent manner (Biteau et al., 2003; Chang et al., 2004). The biological significance of most of these reversible modifications has been amply investigated under physiological and non-physiological conditions (Bechtel and Weerapana, 2017). On the contrary, knowledge about the impact of irreversible Ox-PTMs on cell physiology is quite restricted.

### Reversible Thiol Modification: Addition of Groups to Cysteine

*S-nitrosylation*, the covalent attachment of a nitrous (NO) moiety to –SH functional groups, protects proteins against further cellular oxidative and nitrosative stress. Specific enzymes, called nitrosylases, are responsible for NO group transfer in either metal-to-cysteine or cysteine-to-cysteine mechanisms (Anand and Stamler, 2012). Treatments of mouse hearts with NO donors to increase *S*-nitrosylation, especially in mitochondria, prior to an ischemic insult, reduce indeed infarct size by avoiding critical cysteine irreversible oxidation (Duranski et al., 2005). Under physiological conditions, it represents an important modulator of signal transduction pathways: mitochondrial SNO proteins inhibit respiratory complex I (CI) to modulate mitochondrial ROS production, promote the selective import of mitochondrial protein, enhance mitochondrial fission, and affect the mitochondrial permeability transition pore (MPTP) opening (Piantadosi, 2011; Fernando et al., 2019). However, aging or environmental toxins that increase NO production lead to aberrant *S*-nitrosylation reactions that contribute to neurodegenerative diseases, including Alzheimer’s disease (AD) and Parkinson’s disease (PD) (Nakamura et al., 2013).

*Sulfhydration* (SSH) has lately been acknowledged as a PTM analogous to nitrosylation that consists in the conversion of a –SH group to a –SSH or a persulfide group. Hydrogen sulfide (H_2_S) represents a ubiquitous gaseous signaling molecule with important physiological vasorelaxant properties (Iciek et al., 2015; Zhang et al., 2017) that, in mammals, is enzymatically generated by three enzymes: cystathionine β-synthase (CBS), cystathionine γ-lyase (CTH or CSE), and 3-mercaptopyruvate sulfurtransferase (3MST) (Rose et al., 2017). The reason that proteins undergo this type of modification is not known, although they have been identified by LC–MS/MS. Recently, Tonks et al. suggested that H_2_S, produced by CSE consequently to endoplasmic reticulum (ER) stress, sulfhydrates protein tyrosine phosphatase 1B (PTP1B) (Krishnan et al., 2011). This event, in turn, causes the ER kinase PERK activation during the response to ER stress. Interestingly, anomalous sulfhydration has been linked to several pathological conditions ranging from heart diseases to neurodegeneration (i.e., PD) (Paul and Snyder, 2018).

*S-acylation* is a highly conserved PTM that takes place in all eukaryotic organisms and is regulated by the same enzyme families from yeast to humans. It consists in the covalent attachment of an acyl chain to a cysteine residue and is the only fully reversible posttranslational lipid modification of proteins. Because of the weak nature of the thioester bonds within the intracellular environment, most *S*-acylated proteins rapidly undergo *S*-acylation and deacylation cycles (Chamberlain and Shipston, 2015; Feldman et al., 2015; Pedro et al., 2017). *S*-acylation increases protein hydrophobicity that, in turn, can affect several properties ranging from structure to assembly, maturation, and function. Although the major lipid incorporated into endogenous proteins is palmitate (C 16:0), fatty acids such as oleate (C 18:1) and stearate (C 18:0) can also be added. In particular, *S*-acylation is detected in transmembrane proteins where it is required for stable membrane binding. In this regard, the blockage in the *S*-acylation of specific residues increases protein ubiquitination and degradation (Valdez Taubas and Pelham, 2005). The recent development of *S*-acylation proteomic profiling together with the identification of enzymes that regulate protein *S*-acylation and deacylation has brought new interest into the physiological function of this peculiar Ox-PTM. To date, 47 acylated proteins have been detected in the yeast *Saccharomyces cerevisiae* (Roth et al., 2006) whereas mammalian cells contain hundreds of these modified proteins (Wan et al., 2007; Yang et al., 2010). Interestingly, there is no evidence for *S*-acylation occurrence in prokaryotes. On the contrary, *S*-acylation of many viral proteins is catalyzed by the host cell machinery (Kordyukova et al., 2010).

*S-glutathionylation* (SSG) consists in the addition of the tripeptide glutathione (GSH), the main low-molecular-weight antioxidant of both prokaryotes and eukaryotes, to protein cysteine residues through the establishment of a covalent linkage (Dalle-Donne et al., 2009). This reversible thiol modification is promoted by oxidative and/or nitrosative stress and acts as a repository for reduced glutathione, since the oxidized form (GSSG) is either rapidly excreted from the cells or reduced back to GSH via NADPH-dependent glutathione reductase (Lushchak, 2012). Anyhow, *S*-glutathionylation also occurs under unstressed conditions (Dalle-Donne et al., 2009). Not all cysteines can undergo *S*-glutathionylation: it has been proposed that low p*K*_*a*_ values and, possibly, the three-dimensional proximity to His, Lys, and Arg residues are key factors that make specific Cys appropriate targets for such PTMs (Grek et al., 2013). Numerous molecular mechanisms have been suggested to explain protein *S*-glutathionylation, ranging from the direct thiol–disulfide exchange reaction of a cysteinyl residue with GSSG to the activation of a thiol group to reactive sulfur intermediates (sulfenyl amide, sulfenic acid, or *S*-nitrosyl) which then react with GSH (Allen and Mieyal, 2012). The enzymes responsible for GSH conjugation are called glutathione *S*-transferases, while the reverse reaction is catalyzed by glutaredoxins (Grxs). Certainly, the main role of *S*-glutathionylation is to prevent thiol irreversible oxidation, though it can affect protein function by altering charge and molecular mass, by inducing conformational changes, or by sterically blocking catalytic sites (Grek et al., 2013). The synergy between *S*-glutathionylation and *S*-deglutathionylation thus contributes to the regulation of redox homeostasis, cytoskeletal assembly, protein folding, and stability. Moreover, several evidences have reported their involvement in the control of cell signaling pathways associated with viral infections and apoptosis induced by tumor necrosis factor (Dalle-Donne et al., 2009). Mitochondria contain several *S*-glutathionylation targets thanks to the thiol transferase glutaredoxin 2 (Grx2), which is considered an important redox sensor within the organelle (Xiong et al., 2011).

### Reversible Thiol Modification: Redox Modification of Cysteine Sulfur

*Disulfide bonds* (RS-SR) are essential PTMs involved in protein folding and in the stabilization of their tertiary and quaternary structures. Disulfide formation depends on the spatial proximity to another cysteine and can also occur through a reaction with sulfenic acid in the presence of high concentrations of ROS. They indeed convert SOH groups into thiol radicals (RS⋅) which, in turn, react with other thiolates to form a disulfide bond (reviewed in Summa et al., 2007; Depuydt et al., 2011; Herrmann and Riemer, 2012). In eukaryotic cells, specific enzymes catalyze disulfide exchange within the ER and the mitochondrial intermembrane space (IMS). In yeast, for example, the ER contains the sulfhydryl oxidase endoplasmic oxidoreductin 1 (Ero1) that transfers oxidizing equivalents first to the disulfide isomerase (PDI) and then to secretory proteins. Disulfide formation in the IMS of mitochondria is entrusted, instead, to the MIA system, which will be described in depth in the following sections (Stojanovski et al., 2008). Noteworthy is that some RS-SR are dynamic and modulate protein signaling: that is, in mammalian cells, the stress sensor NPGPx transmits oxidative stress signals through its Cys57–Cys86 disulfide bond. Once oxidized, NPGPx binds to GRP78 (glucose-regulated protein), giving rise to covalent intermediates between NPGPx-Cys86 and GRP78-Cys41/Cys420 that culminate in the enhancement of the GRP78 chaperone activity. The knockout (KO) of NPGPx gene increases the intracellular ROS content and impairs GRP78 chaperone activity, leading to the accumulation of misfolded proteins (Wei et al., 2012). An involvement of S–S bridges in neurodegenerative disorders has been recently proposed. Some evidence suggests that intermolecular disulfide bonds in mutant superoxide dismutase 1 (SOD1) may have a role in its aggregation, a hallmark of some familial amyotrophic lateral sclerosis (ALS) variants (Furukawa et al., 2006). Moreover, intramolecular and intermolecular disulfide bonds seem to be involved in the pathological Tau isoforms typical of AD and other neurodegenerative disorders (Walker et al., 2012; Kim et al., 2015).

*Sulfenylation* (RSOH) is the first step of cysteinyl thiol oxidation. Because of their high reactivity and instability, sulfenic acids (–SOH) have been reputed for a long time as intermediates to additional cysteine modifications (Lo Conte and Carroll, 2013). They can be generated following the condensation of a thiol with a hydroxyl radical, the hydrolysis of *S*-nitrosothiols, and the reaction of a thiol or a thiolate anion with a high concentration of hydrogen peroxide and with thiosulfinates (Hall et al., 2010). Moreover, sulfenic acids can result from the reaction of a thiol or thiolate anion with hypochlorous acid, which represents a powerful bactericidal compound generated within neutrophils during inflammation. The reactivity of the thiol side chain is modulated by the surrounding protein environment that drives the fate of sulfenic acid to an irreversible chemical modification or to a “protective” disulfide bond (Ferrer-Sueta et al., 2011; Roos and Messens, 2011). Accumulating evidence proposes cysteine *S*-sulfenylation as a redox-based signal transduction mechanism in living cells, which is also involved in transcription regulation (Poole and Nelson, 2008; Roos and Messens, 2011; Heppner et al., 2018). The functional domains of several protein phosphatases, acetyltransferases, kinases, deubiquitinases, and deacetylases contain *S*-sulfenylated sites, suggesting the existence of a regulatory cross-talk between *S*-sulfenylation and other major PTM events. Tandem MS revealed –SOH modification also in the catalytic cycle of many enzymes as well as peroxiredoxin (Prx) and NADH peroxidase (Poole et al., 2004; Portillo et al., 2017). Dysregulated protein sulfenylation has been associated with several human pathologies including hypertension (Camargo et al., 2018) and aggressive cancer phenotypes (Truong et al., 2016).

*Sulfinic acid* (RSO_2_H) is the further oxidized step of –SOH achieved in the presence of excess oxidants (Griesser et al., 2018; Urmey and Zondlo, 2020). The biological function of sulfinic acid has been largely debated, due to the general belief that this Ox-PTM could be an artifact triggered by protein isolation procedures. However, this idea was totally discredited after the discovery that the catalytic cysteine of human Prx1 is selectively oxidized to Cys-SO_2_H but not to Cys-SO_3_H, as sulfinic acid is re-reduced to Cys-SH by sulfiredoxin (SRX). In mammals, intracellular sulfinic acid levels depend upon cysteine dioxygenase (CDO), a metabolic enzyme that regulates cysteine homeostasis according to the dietary intakes of sulfur amino acids (Stipanuk et al., 2009). In the last decade, a functional role for –SO_2_H modification has been reported for a growing list of proteins (Lo Conte and Carroll, 2013), including the copper–zinc SOD1, the PD protein DJ-1, and the D-amino acid oxidase (DAO) (Blackinton et al., 2009). Sulfinic acid modulates indeed protein metal binding features: in matrix metalloproteases, the oxidation of a cysteine residue to –SO_2_H activates protease function and is essential for catalytic activity. Despite its association with diseases linked to oxidative stress, including cancer and neurodegenerative disorders, the rationale of sulfinic acid is essentially unknown because of the difficulties of its detection (Neumann et al., 2003; Canet-Avilés et al., 2004). Antibodies against sulfinic acid have limited affinity and specificity. Moreover, MS analysis struggles with the existence of other modifications with the same nominal mass shift, like *S*-sulfhydration (–SSH) (Akter et al., 2018).

### Irreversible Thiol Modification: A Way With No Return

*Sulfonic acid* (RSO_3_H) results from the oxidation of –SO_2_H by strong oxidizers such as ONOOH, H_2_O_2_, or HOX, and it is considered a dead-end product since no biological pathway has been discovered for –SO_3_H reduction. Due to their relative stability compared to the reversible thiol modifications, they can be detected directly by MS, although with some difficulties. Sulfinic and sulfonic acid fragmentations are indeed nearly indistinguishable from the fragmentation patterns of other PTMs and necessitate high-resolution MS instruments to differentiate them (Gaillot et al., 2020). To date, the role of cysteine sulfonic acid in protein function/conformation is still controversial since it is not completely clarified if Cys-SO_3_H can be counted as an oxidative damage or a signaling modification. Literature contains just a few examples in this regard. Lim et al. (2008) proposed that the sulfonic acid formation of yeast Prx1 cysteine in an active site enhances the protein chaperone activity, representing a marker of the cumulative oxidative stress in cells. Lately, Wu et al. (2017) refined this concept, concluding that the two indispensable cysteines of human Prx1, cysteine in the active site (C_*P*_), responsible for peroxide reduction, and resolving cysteine (C_*R*_), which deals with C_*P*_ regeneration, exhibit different propensities to sulfonation. Accordingly, the most frequent overoxidation of human Prx1 hits C_*R*_: sulfonation of Prx1 fosters its activity as a chaperone, preventing proteins from unfolding and irreversible aggregation (Wu et al., 2017). The existence of cysteine residues susceptible to sulfonation appears as an exclusive evolutionary feature of eukaryotic Prxs involved in cell survival during oxidative stress and argues in favor of Cys-SO_3_H as a signaling PTM rather than a simple oxidative damage (Delaunay et al., 2002; Jang et al., 2004; Jarvis et al., 2012; Sobotta et al., 2015). Interestingly, overoxidation of Prxs cysteines follows a precise circadian rhythm in human blood cells that has been identified as a transcription-independent marker (O’Neill and Reddy, 2011; Edgar et al., 2012). The Cys149 residue of the active site of GAPDH is also rapidly overoxidized to sulfonic acid by H_2_O_2_, and this modification is associated with glycolysis inhibition and with numerous side activities including the induction of apoptosis (Colussi et al., 2000). Cys149 irreversible oxidation is regulated by *S*-glutathionylation and disulfide bond formation with the residue Cys154 (Muronetz et al., 2020). In human mutant SOD1, the peroxidation of Cys111 to sulfonic acid has been implied in the pathology of the degeneration/death of familial ALS motor neurons. Immunohistochemical analysis proved indeed the accumulation of mutant SOD1 with overoxidized Cys111 residue in the Lewy body-like hyaline inclusions and vacuole rims of the spinal cord of familial ALS-transgenic mice (Fujiwara et al., 2007). A pathophysiological relevance of sulfonation has been observed also in PD: an increase in the level of sulfonated parkin correlates with its insolubility in human PD brains (Chakraborty et al., 2017). In endocrinology, sulfonation represents a particularly explored biological phenomenon (Wallace et al., 2010). Cys-SO_3_H modification of neuroendocrine peptides strongly affects their receptor binding (Wallace et al., 2010). The oxidation of an N-terminal cysteine residue to sulfonic acid, such as the Cys2 residue of GTPase-activating proteins, has been described as a marker for ubiquitin-dependent protein degradation (Arg/N–degron pathway) (Barros and McStay, 2020). The acetylation of the N-terminal amino group also represents a protective mechanism against irreversible oxidation and seems to act as a degradation signal in the Ac/N–degron pathway. Sulfonic acid is present in mammalian cells as taurine, a low-molecular-weight antioxidant, also an osmoregulator and participant in calcium signaling pathways. Taurine is particularly abundant in the heart, skeletal muscle tissue, retinas, and central nervous system.

## Mitochondrial Proteins PTMs Involving Cysteine Oxidation

Cysteine residues constitute only 2% of the total amino acid content, but the mitochondrial proteome is rich in protein thiols. Mitochondria are dynamic organelles responsible for maintaining redox cellular homeostasis thanks to the continuous generation and wasting of ROS/RNS. The production of ATP and the formation of the proton gradient are, however, imperfect, as the premature leak of electrons from complexes I and III leads to the formation of O_2_^•^^–^. MnSOD rapidly dismutates O_2_^•^^–^ to hydrogen peroxide more stably than other ROSs. The electron transport chain, in addition to the nitrite reductase activity, has been proposed as responsible for the NO⋅ generation within mitochondria: the existence of a mitochondrial nitric oxide synthase (mtNOS) is, indeed, still debated. H_2_O_2_ is the most relevant radical species in redox signaling that reversibly oxidizes protein cysteine thiols (–SH) to sulfenic acid (–SOH) through a mechanism called “redox switch” (Spadaro et al., 2010). However, O_2_^•^^–^ also exerts a signaling role by disassembling Fe–S clusters in several enzymes of the TCA cycle and in respiratory complexes (Mailloux et al., 2014). At high concentrations, O_2_^•^^–^ and H_2_O_2_ can lead to oxidative stress due to the production of the hydroxyl radical (⋅OH), the ROS that is most reactive. In addition, O_2_^•^^–^ can combine with nitric oxide (NO) to form the powerful oxidant peroxynitrite (ONOO^–^) (Fridovich, 1995; Radi, 2013). Under physiological conditions, ROS/RNS generated in mitochondria mainly regulate mitochondrial protein interactions, localization, and activity (Ray et al., 2012; Mailloux et al., 2014) through the oxidative modification of redox-sensitive cysteine residues. It has been reported that the sulfenylation of the Cys253 residue of uncoupling protein 1 (UCP1) within the brown adipose mitochondria of mammalian cells participates in the thermogenic regulation of energy expenditure (Chouchani et al., 2016). *In vivo* in mice, the selective *S*-nitrosation of the Cys39 residue on the ND3 subunit of CI was instead proposed to be cardioprotective (Chouchani et al., 2013). If unregulated, ROS can oxidatively damage biomolecules (proteins, DNA, and lipids), ultimately leading to neurological disorders, metabolic diseases, aging (Bulteau et al., 2006), and other pathologies. Hence, the control of the cellular ROS balance mainly relies on two redox regulatory systems: the reduced/oxidized thioredoxin and the glutathione (GSH)/glutathione disulfide (GSSG) (Holmgren et al., 2005; Jones, 2006).

### Mitochondrial Compartments Possess Different Redox Potentials

The outer mitochondrial membrane (OMM), non-specifically permeable to low-molecular-weight solutes, and the inner mitochondrial membrane (IMM), selectively permeable, define two distinct aqueous compartments within the organelle: the matrix and the IMS. The mitochondrial matrix, that is, the innermost compartment, exhibits a pH of about 8.0 (Llopis et al., 1998) and is the site of numerous enzymatic reactions including organellar DNA replication, transcription, and protein biosynthesis. Components of the mitochondrial IMS are involved, instead, in several processes ranging from the transport of proteins, metal ions, or electrons to the inner membrane proteins assembling up to cellular respiration. The IMS also sequesters several apoptotic components released into the cytosol to trigger programmed cell death. The IMS pH value is about 0.8 units more acidic than the matrix: this asymmetry creates an electrochemical gradient across the inner membrane, exploited in ATP synthesis (Holoubek et al., 2007). Interestingly, the pH of the matrix and IMS differently affects their redox potentials. ROS production is strongly influenced by pH (Shu et al., 1997): in hepatocytes, the extracellular pH controls hydroxyl radical (⋅OH) and NO production (Shu et al., 1997). Likewise, the more acidic pH of the mitochondrial IMS makes it a strong oxidizing environment (Hu et al., 2008; Kojer et al., 2012, 2015). Besides, the IMS of eukaryotic cells contains a dedicated machinery, called the MIA/CHCHD4 complex or disulfide relay-dependent import machinery, responsible for the import and oxidative folding of substrates with conserved cysteine motifs (CX_3_C and CX_9_C). The oxidoreductase Mia40 and the FAD-dependent sulfhydryl oxidase essential for respiration and viability 1 (Erv1) are the crucial components of the MIA complex in yeast. Initially, Mia40 forms a transient intermolecular disulfide bond with imported precursor proteins. Afterward, the isomerization of this disulfide bond causes the introduction of an intramolecular S–S bridge within the imported precursor protein. Following oxidation of the substrate, Mia40 is reoxidized by Erv1 (Lee et al., 2000; Dabir et al., 2007; Banci et al., 2009; Tienson et al., 2009; Bien et al., 2010). A stepwise evolution of the MIA pathway has been proposed in Allen et al. (2008). Accordingly, the ancestral IMS import pathway only included an Erv1 protein, as nowadays in *Leishmania* and *Trypanosoma*. At this stage, Erv1 can still work alone, suggesting that Mia40 behaves as a kind of import chaperone. In fungi, Erv1 necessarily required Mia40 to oxidize substrate proteins because of its inability to do it by itself. The metazoan homolog of yeast Mia40 is the CHCHD4 protein, with the same role as the core component of the disulfide relay-dependent import machinery. They share the same ability to oxidize pairs of reduced cysteines to disulfide bridges, provided that they are present in the motif CX_3_C or CX_9_C. While the yeast Mia40 is a transmembrane protein, metazoan CHCHD4 is smaller, soluble, and moving inside the IMS. Since Mia/CHCHD4 are involved in the proper folding of Cys-containing proteins, they are also considered key components of the quality control system that impacts the IMS targeted proteins (Chatzi and Tokatlidis, 2013). To date, several IMS proteins have been detected with structural disulfide bridges, and the list is constantly increasing (Cavallaro, 2010; Gross et al., 2011; Vögtle et al., 2012). A few examples are the intermitochondrial space Atp23 protein, which is a strongly oxidized protease associated with the IMM (Weckbecker et al., 2012); the protein anamorsin, implicated in Fe/S cluster assembly (Banci et al., 2011); and the Ca^2+^ uniporters MICU1 and MICU2 (Petrungaro et al., 2015). Disulfide bonds within IMS proteins have also lately been considered as part of a quality control system: Longen et al. (2014) proposed that the presence of these Ox-PTMs in the eukaryotic mitochondrial ribosomal Mrp10 protein is sufficient to avoid its proteolytic degradation in the IMS before moving to the mitochondrial matrix. Nevertheless, the presence of special reductases (i.e., thioredoxin and glutaredoxin; Holmgren, 2000) within the IMS causes the reduction of cysteine residues in many proteins and protein domains contained therein. Most of these cysteines are coordination sites for metal ions (Sco1, Sco2, Cox2, Cox11, and Cox17), heme cofactors (cytochrome *c* and cytochrome *c*_1_), and iron–sulfur clusters (Rieske Fe/S protein Rip1). The mitochondrial matrix possesses several redox repair systems: reduced/oxidized thioredoxin and the GSH/GSSG are both involved in disulfide bonds or sulfenic acid reduction (Santo-Domingo et al., 2015).

### Redox Modification of OMM Proteins: The VDAC Case

**FIGURE 1:**
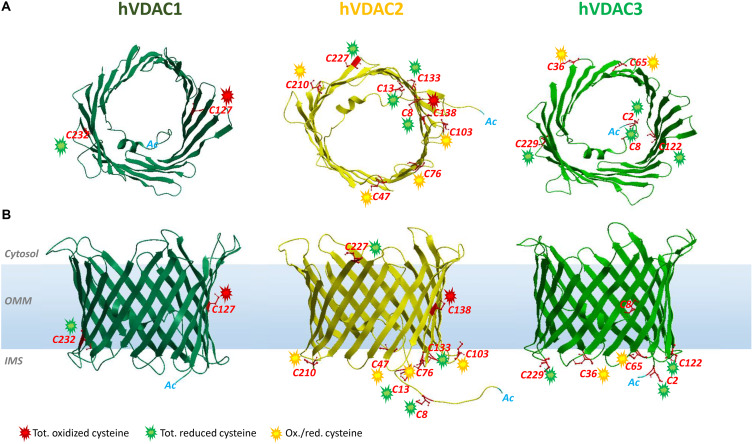
Structure of human VDAC isoforms highlighting cysteine residues and their preferred oxidation states. The structures of hVDAC2 (yellow) and hVDAC3 (light green) β-barrels were modeled using the structure of hVDAC1 [dark green (PDB ID: 2JH4)] as a template. **(A)** Top view of hVDACs with the N-terminal α-helix folded within the pore. **(B)** Side view of the β-barrels embedded in the OMM. The cysteine residues are highlighted (in red), and their oxidative state is indicated by colored sparks: green (totally reduced), red (totally oxidized), or yellow (red/ox, i.e., partially oxidized). There are no VDAC cysteines in the outer face of the membrane, exposed toward the cytosol.

In spite of plenty of reports about the redox modifications of the mitochondrial matrix, IMS, and inner membrane proteins, very little is known about the outer membrane proteins. This is possibly due to underrepresentation of membrane proteins in proteome profiles (protein heterogeneity, hydrophobicity, limited solubility, restricted enzyme accessibility, and low abundance are responsible for it) that downsizes their detection and identification. In order to overcome these restrictions, numerous strategies have been developed to increase the enrichment, solubilization, separation, and cleavage of membrane proteins (Schmitt et al., 2006; Niemann et al., 2013; Bak et al., 2017). Voltage-dependent anion selective channels (VDACs) are surely the most represented proteins of the OMM involved in cellular processes that range from metabolism regulation to cell death control (Benz, 1994; Colombini et al., 1996). Alterations of VDAC expression and activity correlate with several pathologies such as cancer and neurodegenerative disorders (Shoshan-Barmatz et al., 2010; Magrì et al., 2016, 2018; Reina and De Pinto, 2017). In higher eukaryotes, VDAC exists in three different isoforms called VDAC1, VDAC2, and VDAC3 according to the chronological order of their uncovering. Evolutionary analyses suggest that VDAC3 is the oldest protein and VDAC1 is the most recent mitochondrial porin (Young et al., 2007; Messina et al., 2012). Structurally, VDAC forms an aqueous channel arranged in a transmembrane β-barrel linked to an α-helix moiety at the N-terminus (Bayrhuber et al., 2008; Hiller et al., 2008; Ujwal et al., 2008; Schredelseker et al., 2014). In each β-strand, hydrophilic and hydrophobic residues alternate regularly: the former point to the lumen of the pore while the latter interact with the membrane environment. Interestingly, all β-strands follow a steady array of antiparallel units with the exception of β-strands 1 and 19 that, running parallel, make both the N-terminus and the C-terminus point toward the IMS (Tomasello et al., 2013). The amphipathic α-helix tail seems to be placed inside the pore, but, to date, the exact position and local structure of this segment are still debated (De Pinto et al., 2007). However, although the general organization of the three isoforms is similar, different molecular weights and amino acid compositions may be associated to distinct roles in the cell. In particular, VDAC isoforms differ in the number and distribution of cysteines so that they have been the subject of specific investigations to unravel a peculiar role for these residues in protein activities (De Pinto et al., 2016; Reina et al., 2016). In humans, VDAC1, VDAC2, and VDAC3 have two, nine, and six cysteines, respectively. Considering VDAC3 is the oldest isoform, it is tempting to speculate that evolution has decreased cysteine number in VDAC1, according to an ubiquitous expression and proapoptotic function, while increasing cysteine content in VDAC2, possibly to favor its involvement as an antiapoptotic protein (De Pinto et al., 2016). VDACs are exposed to both cytosol and IMS (Figure 1). Interestingly, many of the cysteine residues are located in protein loops exposed to IMS and its oxidative potential. In addition, VDAC has been proposed as the only escape route from the mitochondria of the unreacted superoxide radical (O^2–•^), a by-product of complex III of the respiratory chain (Han et al., 2003). At a variance from hydrogen peroxide (H_2_O_2_), O^2–•^ is very active but has a short life and cannot freely diffuse trough the membrane (Bleier et al., 2015). Our research group was the first to report a detailed profile of the oxidative state of VDAC cysteines and methionines (Saletti et al., 2017, 2018) by affinity chromatography purification and UHLC/high-resolution *n*-ESI-MS/MS. What we noticed is that a cysteine redox modification pattern is conserved during evolution. In both human (Pittalà et al., 2020) and rat VDACs (Saletti et al., 2017, 2018), indeed, residues are in the reduced form (i.e., free thiol groups available to disulfide bond formation), while others are constantly and irreversibly overoxidized to sulfonic acid. We thus hypothesized that a specific modification of each cysteine may correlate to a specific structural or functional role. Very recently, a “shot-gun” cysteine-targeted MS analysis that identified ∼1500 reactive cysteine residues on ∼450 mitochondrial proteins in HEK293T cells was able to detect cysteines highly sensitive to *S*-nitrosoglutathione (GSNO) also in VDAC proteins (Bak et al., 2017).

### Cysteine Residues in VDAC Isoforms

#### VDAC1

VDAC1 is the best-characterized VDAC isoform. It provided the first description of typical voltage dependence and ion selectivity features that are the fingerprint of these pores. VDAC1 has been thoroughly characterized by electrophysiological studies (Blachly-Dyson et al., 1993; Xu et al., 1999) and is considered one of the main actors of bioenergetic metabolism and cell death regulation (Maldonado et al., 2010; Camara et al., 2011; Maldonado and Lemasters, 2012). Its 3D structure was elucidated (Bayrhuber et al., 2008; Hiller et al., 2008; Ujwal et al., 2008). A large number of reports describe VDAC1 as a docking site for proapoptotic (Bax, Bak, and Bim) and antiapoptotic (Bcl2 and Bcl-xL) factors (Shimizu et al., 2001; Vander Heiden et al., 2001; Arbel et al., 2012; Huang et al., 2013; Liu et al., 2015), as well as for cytosolic enzymes [hexokinases (HK) I and II] (Fiek et al., 1982; Abu-Hamad et al., 2008). The interaction with HK makes VDAC1 a key player in cancer and other pathologies, including neurodegenerative diseases (Maldonado and Lemasters, 2012; Leanza et al., 2014; Reina and De Pinto, 2017; Magrì et al., 2018), that share mitochondrial dysfunction and oxidative stress. VDAC1 contains only two cysteines (Cys127 and Cys232). Cys127 residue (β-strand 8) protrudes in phospholipidic hydrophobic milieu, while Cys232 is located in β-strand 16 and faces the water-accessible side of the channel (Bayrhuber et al., 2008; Hiller et al., 2008; Ujwal et al., 2008). Still, there is no evidence that oxidation can rule VDAC1 activity through the Ox-PTMs of its sulfhydryl groups. Nevertheless, already in De Pinto et al. (1991), the authors established that the two conserved cysteine residues of VDAC1 from bovine heart mitochondria are sensitive to oxidation. Both the oxidized and reduced forms of VDAC1, however, showed the same biophysical properties into artificial lipid membranes, disclaiming any redox regulation of channel activity (De Pinto et al., 1991). Later, Aram et al. (2010) confirmed that the pore is not affected by VDAC1 cysteine deletion and observed that ROS-producing agents such as selenite, As_2_O_3_, or H_2_O_2_ do not change apoptosis in human cells overexpressing rat VDAC1. Moreover, cross-linking experiments let them conclude that VDAC1 cysteines are not involved in intermolecular S–S bridge and thus do not take part in oligomerization (Aram et al., 2010). Overall, reports available so far agree that cysteine residues have no functional role in VDAC1 (Teijido et al., 2012). Recent findings revealed a conserved pattern in oxidative status, conserved among human and rat VDAC1: Cys127 was always detected in the trioxidized form of –SO_3_H and Cys232 exclusively in the reduced and carboxyamidomethylated form (Pittalà et al., 2020).

#### VDAC2

**FIGURE 2:**
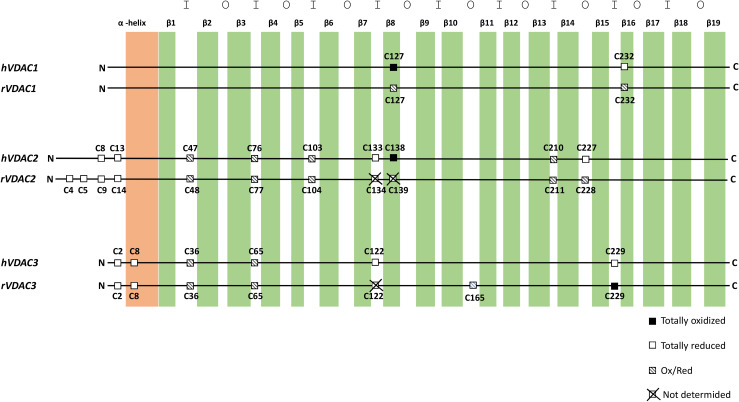
Cysteine localization in the aligned sequences of human and rat VDAC isoforms. The N-terminal α-helix is shown in light orange, and β-strands are in green. The internal loops, that is, exposed to the intermembrane space, are indicated with I. The outer loops, that is, exposed to the cytosol, are indicated with O.

Analogous to isoform 1, VDAC2 participates in several cellular processes, from metabolite exchange through the OMM (Maurya and Mahalakshmi, 2016; Naghdi and Hajnóczky, 2016) to calcium homeostasis regulation (Subedi et al., 2011; Shimizu et al., 2015). Although ubiquitous, it is predominantly expressed in the heart and testes and, at the same time, poorly represented in the liver (Yamamoto et al., 2006; Calvo et al., 2016). The N-terminal region, which contains the voltage sensor of the channel, carries an extension of 11 additional residues only in VDAC2. The only available crystal structure is zebrafish VDAC2 (zfVDAC2), whose sequence is only about 90% similar to mammalian VDAC2 (Schredelseker et al., 2014), and confirms substantial analogy with the published structures of mouse and human VDAC1. Mouse, human, zebrafish, and bovine isoform 2 easily insert into bilayer membranes as canonical VDAC pores with similar single-channel conductance, voltage gating, and anion selectivity (Blachly-Dyson et al., 1993; Xu et al., 1999; Komarov et al., 2005; Menzel et al., 2009; Maurya and Mahalakshmi, 2015). VDAC2 is considered antiapoptotic, a distinction from VDAC1 and VDAC3: VDAC2 complexes with the proapoptotic factors BAK (Cheng et al., 2003) and BAX (Chin et al., 2018), thereby hampering programmed cell death. Accordingly, VDAC2 KO is lethal *in utero* (Cheng et al., 2003), and conditional KO animals display severe dysfunction in the apoptotic pathways (Ren et al., 2009). VDAC isoform 2 also possesses the highest cysteine content (nine in humans and 11 in rats, Figure 2). This intriguing hallmark has prompted numerous studies aimed at identifying the role of such residues in protein structure and function. Maurya and Mahalakshmi (2013, 2014, 2015) demonstrated that VDAC2 cysteines strengthen β-barrel association to OMM, while lowering structure stability. Moreover, it has been reported that deletion (Majumder and Fisk, 2013) or modification (Piroli et al., 2016) of human VDAC2 cysteine residues can impair channel function. For instance, VDAC2 Cys47 and Cys76 succination has been linked to reduced ATP synthesis within mitochondria of the Leigh syndrome mouse model (Piroli et al., 2016). In Naghdi et al. (2015), cysteines are considered unessential for VDAC2-mediated Bak recruitment and tBid−induced cytochrome *c* release. A role for cysteines in ROS regulation has also been speculated (Maurya and Mahalakshmi, 2015), although no experimental evidence has yet proven it. Saletti et al. (2018) and Pittalà et al. (2020) first supplied a detailed profile of VDAC2 Cys Ox-PTMs that reveals a precise and evolutively conserved scheme in thiol oxidation. The N-terminal cysteine residues 8 and 13, together with the unique thiol group located in a loop exposed toward the cytosol (i.e., Cys 227 in Figure 1), were reported as totally reduced. Cysteines 47, 76, 103, and 210, all located in IMS loops, were instead identified as partially oxidated to –SO_3_H. Surprisingly, VDAC2 Cys138, which faces the lipid environment of the OMM (Figure 1), was found fully trioxidized to sulfonic acid as the homologous residue in VDAC1 (Cys127, Figures 1, 2).

#### VDAC3

VDAC3 is the least known isoform. Its 3D structure has not yet been experimentally resolved, albeit several homology modeling predictions have confirmed a β-barrel organization almost identical to VDAC1 and VDAC2 (Bayrhuber et al., 2008; Hiller et al., 2008; Ujwal et al., 2008; De Pinto et al., 2010; Schredelseker et al., 2014). Unlike VDAC1 and VDAC2, ubiquitously expressed in all eukaryotes, VDAC3 prevails in the cerebral cortex, liver, heart, testes, and spermatozoa (Sampson et al., 2001). Herein, it participates in sperm mobility since VDAC3-null mice show a single microtubule doublet loss within the epididymal axoneme (Sampson et al., 2001) and in disassembly of cilia during the cell cycle by targeting Mps1 protein kinase to centrosomes (Majumder and Fisk, 2013). *S. cerevisiae* cells devoid of endogenous porin (Δ*por1*) have been extensively exploited for functional studies on VDAC proteins: as reported, the heterologous expressions of VDAC1 and VDAC2 fully complement the growth defect associated with gene loss, while that of VDAC3 only partially did so (Sampson et al., 1997; Reina et al., 2010). VDAC3 pore-forming activity was more difficult to be detected and did not show the homogeneous activity usually found with VDAC1 and VDAC2. Recombinant VDAC3 resulted in poor insertion in bilayers and hectic pore formation (Xu et al., 1999; Checchetto et al., 2014). In Checchetto et al. (2014), we demonstrated that, under non-reducing conditions, the majority of inserted VDAC3 forms very small pores with a lower conductance compared to the other isoforms. The modified activity pattern of VDAC3 pushed to look for differences between the VDAC3 structure and, in particular, VDAC1. The most striking was the cysteine content (De Pinto et al., 2016). Human VDAC3 contains indeed six cysteines (residues 2, 8, 36, 65, 122, and 229; Cys2 is the actual N-terminal residue, since Met1 is removed after biosynthesis) (Saletti et al., 2017, 2018; Pittalà et al., 2020). Except for Cys8, located within the pore, all cysteine residues protrude toward the IMS. The first clue about the relevance of VDAC3 cysteines goes back to the year 2010, when “swapping” experiments of the VDAC3 N-terminus (containing two cysteines) with the corresponding region of VDAC1 (lacking cysteines) totally changed isoform 3 pore activity (Reina et al., 2010). Next, electrophysiological data reported VDAC3 as able to form typical voltage-dependent pores (although with a remarkable lower insertion rate compared to VDAC1 and VDAC2), when refolded under highly reducing conditions (Queralt-Martín et al., 2020). Deletion of cysteines in engineered human VDAC3 molecules strongly affected both electrophysiological parameters and its ability to revert the mutant phenotype of Δpor1 yeast (Okazaki et al., 2015; Reina et al., 2016). The most relevant residue(s) for protein function appears to be 2, 8, and 122 in human VDAC3, since their mutation always restored formation of large pores and Δpor1 growth (Reina et al., 2016). Furthermore, the simultaneous mutation of any VDAC3 cysteines to alanine had a similar effect (Queralt-Martín et al., 2020). From MS analysis (Saletti et al., 2018; Pittalà et al., 2020) the preferred redox state of cysteines is conserved between rat and human VDAC3 analyzed. Accordingly, Cys2, Cys8, Cys122, and Cys229 were entirely identified in the reduced form, with the N-terminal Cys2 found acetylated. Cys36 and Cys65 were detected in a reduced form and trioxidized to sulfonic acid.

## Conclusion

### Hypotheses About the Role of Cys Ox-PTMs in VDAC Isoforms

Based on MS data reported for VDAC1, VDAC2, and VDAC3 in humans and rats, we found that the oxidation state of each specific residue is the same (Saletti et al., 2018; Pittalà et al., 2020). Thus, we speculated about the relationships between location of residue and oxidation state. In Figure 1, the position of the cysteines with respect to the water/phospholipidic phases is shown. Figure 2, instead, exhibits, on an analytical scheme, the oxidative modifications borne by the residues with respect to their location in structural elements of the pore. We thus noticed that cysteines whose lateral residue is embedded in the hydrophobic moiety of the membrane are trioxidized to –SO_3_H. This finding is very surprising, since the charged modified sulfur residues protrude into the most hydrophobic part of the membrane bilayer, an apparent oddity, if it has no reason to be, unless it has a function as a spot for a structure intended to dock the OMM. These residues are present only in VDAC1 and VDAC2.

Cysteines in the N-terminal domain sequence steadily emerge as totally reduced. To be detected in MS as carboxymethylated sulfur, they indeed have to be protected *in vivo* by irreversible oxidation events. VDAC2 and VDAC3 alone have cysteines in the N-terminus. This group of totally reduced cysteines, exposed to IMS or residing inside the channel, could form disulfide bonds in VDACs as proposed in Okazaki et al. (2015) and Reina et al. (2016) and with the aim of changing pore useful diameter for modulating water-soluble molecule flow. It is not known how the formation of these hypothetical disulfide bridges may happen. It is tempting to speculate that the MIA/CHCHD4 complex could be involved. Although VDACs cannot be the subject or component of this specific quality control system because of a different import pathway, it is suggested to imagine that in metazoans CHCHD4 might interact with the VDAC2 and VDAC3 cysteines protruding toward IMS in reduced form (Pittalà et al., 2020). VDAC2 shows a motif CX_4_C and VDAC3 a motif CX_5_C in the N-terminal end. The possibility that CHCHD4 can oxidize cysteine pairs in a non-conventional motif has been highlighted for mitochondrial p53, which contains CX_5_C and CX_1_C motifs (Zhuang et al., 2013; Park et al., 2016), and it is true also for Erv1, containing a CX_16_C motif (Terziyska et al., 2007). CHCHD4, indeed, could represent a direct link of VDAC with apoptosis-inducing factor (AIF) (reviewed in Reinhardt et al., 2020): CHCHD4 is the first AIF mitochondrial interactor that was discovered. A CHCHD4 cysteine pair is reoxidized by ALR and docked to AIF-NADH. Another intriguing connection between VDACs and AIF could be found in the essential role of AIF in import and folding of CI subunits (Vahsen et al., 2004): it is known that CI is the most important ROS producer within the IMS, and these ROS are candidate to oxidize VDAC cysteines.

A ring of residues exposed or predicted to be exposed to the IMS is the third group of cysteines with distinct modifications as found by MS spectra: it turns out that they are in either oxidized or carboxyamidomethylated form, thus inclined to variable and possible reversible oxidations. This girdle of cysteine residues located in the turns connecting the β-strands and exposed to IMS can thus work as an oxidation buffer located on the inner surface of the OMM with the function of counteracting excess of ROS produced by the OXPHOS (Reina et al., 2016).

Our interpretation of such differences, experimentally determined by MS, is that types of cysteine residues may have specific roles in the operation of VDAC isoforms.

Thus, the pattern of cysteine oxidation could also be the key to understanding the evolution of the three VDAC isoforms: VDAC1 has only two, one of them in sulfonic state and exposed to the hydrophobic layer of the OMM; VDAC2 has 9–11 cysteines, in the three categories we described above; thus, it has also the overoxidized one; VDAC3 has again six to seven cysteines but lacks the category of overoxidized one.

Okazaki et al. (2015) proposed that the transient formation of a S–S bridge between VDAC3 residues placed in the protein N-terminus with those at the bottom of the pore could change its permeability, albeit the nature of such conductance hindrance is not clear yet. Molecular dynamic simulations have shown that disulfide formation between the nearest cysteines available did not affect the channel diameter but rather changed the electrical charge disposition on the protein surface (Amodeo et al., 2014; Guardiani et al., 2016). There are only indications of experimentally determined disulfide bridges in VDAC (Reina et al., 2016). Interestingly, the insertion of negative charges by –SO_3_H formation can modify the protein conformation by electric repulsions inside the chain or toward phospholipids. Some authors suggested that these conformational changes can initiate protein incorporation into mitochondria-derived vesicles (MDVs), later targeted to lysosomes. MDVs, whose production is induced by mitochondrial stress (Sugiura et al., 2014), contain numerous oxidized proteins derived mainly from the OMM. VDAC1 appears among these proteins (Soubannier et al., 2012), whereas no evidence about the VDAC3 cysteine status or its presence within MDVs has been reported.

Posttranslational removal of N-terminal methionine leaves VDAC3 Cys2 as the first amino acid. All proteomic analysis revealed this residue as completely acetylated, a PTM that, beside significantly influencing protein stability, activity, folding, and localization, triggers specific protein degradation by the Ac/N–degron pathway. This peculiar branch of the N-rule pathway controls several aspects of protein quality control (Eldeeb and Fahlman, 2016). Any dysregulation of N-terminal acetylation, which protects cysteine against irreversible oxidation, leads to serious pathological conditions including neurodegenerative diseases, cancers, hypertension, and X-linked genetic disorders (Rope et al., 2011; Aksnes et al., 2015, 2016).

### Possible Pathological Implications of Redox Cysteine Modifications in VDACs

Despite VDAC being involved in several mitochondria-associated pathologies such as hypertension (Alvira et al., 2012; Zhu et al., 2019), diabetes (Sasaki et al., 2012; Zhang et al., 2019), cancer (Maldonado and Lemasters, 2012; Reina and De Pinto, 2017; Magrì et al., 2018), cardiovascular diseases (Karachitos et al., 2017), and neurodegenerative disorders, such as ALS (Magrì et al., 2016), PD (Rostovtseva et al., 2015), and AD (Manczak and Reddy, 2012), very little is known about the correlation of its Cys-Ox-PTMs with the aforementioned diseases. This is undoubtedly attributable to the few data available on these VDAC modifications, whose identification and analysis results are challenging. To quote some examples, Zahid et al. reported alterations in the *S*-nitrosylation pattern of VDAC2 in AD (Zhao et al., 2015); in Lam et al. (2010), the authors listed VDAC1, VDAC2, and VDAC3 among the *S*-nitrosylated targets in the prostate epithelial cell line.

The role of VDAC cysteine disulfide bridges or of its oxidation to sulfonic acid has yet to be discovered. It is suggested that the degree of VDAC oxidation on the mitochondrial surface could function as a signal of ROS load within the mitochondrial network, even if much more needs to be done to truly understand their biological meaning.

## Author Contributions

SR conceived the review organization and wrote most of it. MP selected the references and contributed to the oxidized cysteine discovery. FG, AM, and VD wrote the discussion section. SF and RS reviewed and revised the text and the Mass Spectroscopy analysis.

## Conflict of Interest

The authors declare that the research was conducted in the absence of any commercial or financial relationships that could be construed as a potential conflict of interest.
